# Adult–adult play in captive lowland gorillas (*Gorilla gorilla gorilla*)

**DOI:** 10.1007/s10329-022-00973-7

**Published:** 2022-02-22

**Authors:** Giada Cordoni, Luca Pirarba, Stéphanie Elies, Elisa Demuru, Jean-Pascal Guéry, Ivan Norscia

**Affiliations:** 1 Department of Life Sciences and System Biology, University of Torino, 10123 Torino, Italy; 2 La Vallée des Singes, 86700 Romagne, France; 3 Laboratoire Dynamique Du Langage, CNRS UMR5596, University of Lyon 2, Lyon, France; 4 Equipe de Neuro-Ethologie Sensorielle, ENES/CRNL, CNRS UMR 5292, Inserm UMR S 1028, University of Lyon/Saint-Etienne, Saint-Etienne, France

**Keywords:** Adult play, Oxytocin effect, Play motivation, Role reversal, Play face, *Gorilla gorilla gorilla*

## Abstract

Among African great apes, play is virtually absent between adult lowland gorillas (*Gorilla gorilla gorilla*). Here, we report an extremely rare case of adult–adult play observed in the lowland gorilla group housed at La Vallée de Singes (France). We recorded three playful interactions between the silverback and an adult lactating female. Given the diverse causal and functional nature of play, different factors may join in promoting this behaviour. In our group, contrary to what has been shown by previous studies in wild and captive gorillas, adult females spent more time in spatial proximity with the silverback than with other females. Hence, the probability of social interaction (including play) between silverback and adult females was enhanced. Moreover, the motivation of the lactating female to play might be an effect of oxytocin, a hormone that reaches high concentration levels during lactation and that promotes social affiliation. The fact that play invitations were always performed by the female might support this hypothesis. Certainly, we cannot exclude the possibility that the play event is a group idiosyncrasy or an artefact of captivity, even though the subjects never showed abnormal behaviour. Structurally, play sessions showed a suitable degree of pattern variability and switching frequency from one pattern to another. The proportion of offensive patterns was higher in the female during play and in the male during aggression, which conforms to the role reversal play phenomenon. In conclusion, this report confirms that the absence of evidence is not the evidence of absence. It is likely that under particular physiological or socio-ecological conditions, adult–adult play may be manifested as an “unconventional” part of gorilla social behaviour.

**Supplementary Information:**

The online version contains supplementary material available at 10.1007/s10329-022-00973-7.

## Introduction

In human and non-human primates, social play is widespread among immature individuals and is considered the main social interaction characterizing the juvenile developmental phase (Fairbanks 2000; Cordoni and Palagi 2011). Conversely, adult–adult play has been described in only about 50% of primate species (Pellis and Iwaniuk 1999, 2000). Following a phylogenetic logistic regression analysis, O’Meara and colleagues (2015) could not single out any correlations between the maintenance of play in adulthood and metabolic (e.g. basal metabolic rate), socio-ecological (e.g. group size) or life-history (e.g. age at sexual maturity) variables. This finding supports the diverse causal and functional nature of adult social play behaviour in primates.

African great apes show frequent and quite stable levels of social play in the immature phase (Fagen 1981; Palagi et al. 2007; Cordoni and Palagi 2011; Palagi and Cordoni 2012; Cordoni et al. 2018), but the level of play during adulthood varies greatly depending on the species. In bonobos (*Pan paniscus*), adult–adult play remains relatively frequent across different sex/age-class combinations and contexts (Palagi et al. 2006; Palagi and Paoli 2007; Palagi and Cordoni 2012). In chimpanzees (*Pan troglodytes*), adult–adult playful interactions are less frequent compared to bonobos, although they can still be observed as a means for reducing social tension and strengthening affiliative bonds (Palagi et al. 2004; Cordoni and Palagi 2011; Yamanashi et al. 2018). Although infrequent, social play in adult mountain gorillas (*Gorilla beringei beringei*) has mainly been reported between mature males living in multi-male or bachelor groups (Yamagiwa 1992; Watts and Pusey 1993; Grueter et al. 2016). In lowland gorillas (*Gorilla gorilla gorilla*), social play plummets when approaching sexual maturity and is virtually absent between adults (Stewart and Harcourt 1987; Palagi et al. 2007, 2019; Masi et al. 2009; Cordoni et al. 2018).

Here, we report and describe a rare case of adult–adult play in captive lowland gorillas that was observed between the silverback and an adult female. In the wild, lowland gorillas mainly constitute one-male units comprising an adult male (the silverback), several adult females and their offspring (Fleagle 2013). Mature males can temporarily associate in the so-called bachelor groups during the period of dispersion from their natal groups (Robbins and Robbins 2018; Forcina et al. 2019; Hagemann et al. 2019). Before their first reproductive event, females also disperse from their original groups, and they preferentially join smaller groups led by younger and stronger silverbacks (Stokes 2004; Manguette et al. 2020). Even if affinitive interactions may be present at variable levels in lowland gorillas (Forcina et al. 2019; Cooksey et al. 2020), social contacts (e.g. allo-grooming) between adults are less frequent compared to other great apes (Stokes 2004; Cordoni and Palagi 2007; Masi et al. 2009; Cordoni et al. 2018). In the wild, Stokes (2004) reported only eight affiliative events (i.e. sexual interactions and contact sitting) between adults during 802 hours of observation; intriguingly, six out of eight contacts occurred between the silverback and the reproductive females. In both captive and wild gorillas, first post-conflict affinitive contacts between former opponents (reconciliation sensu de Waal and Roosmalen 1979) occurred more frequently between the alpha male and adult females compared to other dyads, possibly aimed at restoring a peaceful relationship between these subjects (Watts 1995, 2000; Cords and Aureli 2000; Cordoni et al. 2006). Different results have been obtained for spatial proximity (Watts 1994; Stokes 2004; Lemasson et al. 2018). Indeed, in captive lowland gorillas, it has been observed that adult females (particularly females with newborns) did not maintain close proximity with the silverback, and they even preferred staying in spatial closeness with other group members (Fischer 1983; Nakamichi and Kato 2001; Stoinski et al. 2003). As concerns play, in two captive groups housed at the ZooParc de Beauval (France), Cordoni and co-authors (2018) reported extremely low frequencies of playful interactions between adults and immature subjects (mean individual hourly frequency = 0.023 ± 0.015SE), and social play was never observed between adults. In the wild (Odzala-Kokoua National Park; Congo), Forcina and colleagues (2019) qualitatively reported playful interactions between adult females of different groups, but never between adult females and the silverback.

Given the extremely rare occurrence of adult–adult play in lowland gorillas, very little is known about how adults engage in and manage playful interactions. In this report, we structurally describe the social play observed between the silverback and a lactating female, as a starting point for future research on modality, complexity and possible function/s of play in adult gorillas. Due to the complex nature of play, different factors, not mutually exclusive, may join in promoting adult–adult play, such as inter-individual closeness (e.g. spatial proximity), physiological conditions (e.g. lactating period) and individual characteristics (e.g. play propensity). We discuss different scenarios in the possible hypotheses explaining the occurrence of this playful event.

## Study site and subjects

The study was carried out on the family group of lowland gorillas (*Gorilla gorilla gorilla*) housed at La Vallée des Singes (Romagne, France). The group comprised 10 individuals (see Table 1). The silverback sired all immature subjects. Only one adult female (Virunga) in the group had no offspring. During the observations, the primiparous female Mahmah was lactating. The gorilla enclosure included both an indoor and an outdoor facility of about 150 and 3400 m^2^, respectively. The outdoor space was a natural wooded island surrounded by a water canal, and the indoor enclosure was enriched with lianas, trunks, straw and platforms. Gorillas were fed outdoors with fruit and vegetables five times per day during spring and summer (May–Aug: 11:15, 14:00, 15:30, 17:00 and 18:00) and twice per day starting from September (Sep–Apr: 11:15 and 15:30). Water was provided ad libitum. Gorillas were free to move between the indoor and outdoor enclosures and to socially interact. No aberrant or stereotypic behaviour was observed.

**Table 1:** The composition of the lowland gorilla family group hosted at La Vallée des Singes (Romagne, France)

Subject	Sex	Age class and year of birth	Age (years) at the date when adult–adult play sessions occurred (27/09/2020)	Kinship	Year of arrival at La Vallée des Singes
Yaoundé* (YA)	M	Ad—1983	37	MW, DJ, KO, IV and BA father	1998
Hakuna (HA)	F	Ad—1996	24	IV mother	2015
Virunga (VI)	F	Ad—1970	50	No offspring	1998
Moseka (MO)	F	Ad—1984	35	MW, DJ and KO mother	1999
Mahmah* (MA)	F	Ad—2002	18	BA mother	2014
Mawete (MW)	M	SubAd—2011	9	DJ and KO brother	–
Djomo (DJ)	M	SubAd—2008	12	MW and KO brother	–
Kouam (KO)	M	Inf—2016	4	MW and DJ brother	–
Ivindo (IV)	F	Inf—2017	3	–	–
Basoko (BA)	M	Inf—16/05/2020	0	–	–

*M* male, *F* female, *Ad* adult (female > 8 years; male > 12 years), *SubAd* sub-adult (female 6–8 years; male 6–12 years), *Inf* infant (0–4 years). The play sessions that are the focus of this study were observed between the two individuals marked with asterisks

## Data collection and operational definitions

We collected video data during August–October 2020 and March–June 2021 on a daily basis spanning morning and afternoon and including all feeding times. The videos were recorded by one author (SE) using a Panasonic HDC-SD9 video camera. In total, about 72.5 hours of videos were collected. The videos were analysed—also through a frame-by-frame analysis when necessary—via the freeware Avidemux 2.7.8. Before starting the video analysis, GC supervised LP in a training period of 15 hours to reach suitable agreement in individual gorilla recognition and in the classification of behavioural patterns and facial expressions. The interobserver reliability reached Cohen's κ value of 0.78 for individual recognition and 0.74 for behaviour classification (considered behaviours: play, playful facial expressions, aggression, affiliation).

Following Altmann (1974), we employed scan sampling for evaluating baseline activities and spatial distance between subjects (i.e. resting, moving, feeding, spatial proximity, body contact, grooming, play, aggression). Spatial proximity was defined as an arm-length distance between two gorillas. We carried out group scans at 10-minute intervals by collecting a total of 132 scans (22 hours of observation). We also employed all occurrences sampling for recording playful/aggressive events (50.5 hours of observation).

For each playful/aggressive event we reported (i) the identities of the interacting subjects (age and sex) and which of them initiated the session, (ii) behavioural patterns performed in their chronological order (see Table 2 for definition of behavioural patterns), and only for play, (iii) facial expressions (play face, PF; full play face, FPF; see Table 2) and their duration (centiseconds) and (iv) session duration (seconds).

**Table 2:** Play and aggressive behavioural patterns of lowland gorillas considered in this study

Play patterns	Definition
Acrobatic play^N^	The gorilla swings hanging/jumps from a support and makes somersaults/pirouettes in a solitary or social manner
Airplane^N^	The older gorilla holds the smaller playmate with hands/feet above her/his head while lying on the ground
Attempt play bite^O^	The gorilla unsuccessfully tries to close her/his mouth on the partner’s body
Full play face	The gorilla opens her/his mouth with both upper and lower teeth exposed
Peek a boo^O^	The gorilla hides and suddenly pops out from a shelter
Pirouetting^N^	The gorilla performs somersaults/pirouettes on herself/himself or hanging from a rope. Pirouetting can be a part of acrobatic play
Play bite^O^	The gorilla closes her/his mouth on the partner’s body in a non-harmful way
Play brusque rush^O^	The gorilla jumps with her/his four limbs on the playmate generally in a quadrupedal position and either bounces away or stays for initiating a play session
Play carry^N^	The gorilla dorsally or ventrally carries the playmate (typical of play mothering). The carried subject lies on the carrier's back or he/she is in contact with the carrier's lower abdomen in a sort of embrace. The carrier moves
Play climb or stand on another^O^	The gorilla climbs or stands on the playmate’s body independently of the position of the playmate (sitting, lying or standing)
Play drag^O^	The gorilla hauls the playmate taking her/him from the limbs
Play eye cover^O^	The gorilla covers the eyes of the playmate
Play face	The gorilla opens her/his mouth with only the lower teeth exposed
Play “give me five”^N^	Two gorillas interact face-to-face and slap each other’s palms
Play grab^N^	The gorilla gently massages the playmate, holding her/him tightly
Play invitation	The gorilla performs one or more playful behavioural patterns for inviting the fellow to play
Play jump^O^	The gorilla gently jumps alone or on the playmate only with feet generally in a quite bipedal position. Play jumps are generally small, mainly stationary, with little or no forward movement
Play kick^O^	The gorilla gently uses her/his feet to hit the playmate
Play manipulation^N^	The gorilla takes and explores an object without using it for any specific goal
Play moon walk^N^	The gorilla walks backward, generally keeping her/his eyes fixed on the playmate
Play pat^N^	The gorilla repeatedly and gently touches the partner’s body with the palm of her/his hand
Play piggy back ride^N^	The gorilla is placed with a leg on each side of the back of the playmate and his/her torso is erect. This position looks like that of horse rider. The carrier may or may not move
Play pull^O^	The gorilla moves the playmate towards her/him with hands/feet during play
Play push^O^	The gorilla displaces the playmate far from her/him with hands/feet
Play retrieve^O^	The gorilla blocks the playmate with her/his hands to prevent her/his flight. It is different from play pull, which is generally performed with both feet and hands during play
Play roll^N^	The gorilla turns its body from side to side while supine
Play run^O/D^	The gorilla runs alone (solitary play) or behind the playmate (social play) by often changing her/his direction and the playmate runs far from the partner
Play shake the rope^N^	The gorilla forcefully moves the rope on which the playmate is hanging
Play shelter^D^	The gorilla protects herself/himself from playmate slaps, bites, etc., by putting its arms over its head
Play slap^O^	The gorilla uses her/his open hands for hitting any part of the playmate’s body
Play slide down^N^	The gorilla slides down from hill, tree, rocks or other equipment
Play stamp^O^	The gorilla hits on the ground or on the playmate with her/his feet in a repeated way
Play tug-of-war^O^	The gorillas contend an object and pull it toward themselves
Play turn around^N^	The gorilla runs/walks alone around an object without changing her/his direction
Play walk^N^	The gorilla follows the playmate or goes back and forth
Play wriggle^D^	The gorilla wriggles to get free from the grip of the playmate
Rough &Tumble^N^	The gorillas play in tight and continuous physical contact by employing many of the patterns described in this table (e.g. bite, kick, slap, stamp)
Somersault^N^	The gorilla flips over the ground or on vertical supports in solitary or social manner
Tickle^N^	The gorilla tickles with hands/feet any part of the partner’s body

Aggressive patterns	Definition
Aggressive bite^O^	The gorilla encloses a part of the partner’s body with the mouth in a harmful way
Aggressive chest beat^O^	The gorilla usually rises in bipedal position and rapidly beats her/his chest with cupped hands in rapid succession, producing an impressive drumming sound. This behaviour is sometimes preceded by a hooting vocalization
Aggressive leg kick^O^	The gorilla strikes air with foot at the end of a charge or chest beating. It is a threatening behaviour
Aggressive object slapping/stamping^O^	The gorilla hits objects/ground of her/his environment with open hands or feet. It is a threatening behaviour
Aggressive pull^O^	The gorilla aggressively moves the opponent towards her/him with hands/feet
Aggressive push^O^	The gorilla aggressively displaces the opponent far from her/him with hands/feet
Aggressive slap^O^	The gorilla uses her/his open hands for aggressively hitting any part of the opponent’s body
Avoid^D^	The gorilla moves out of the path when another individual is approaching her/him or takes a less direct route around the other
Bared-teeth^N^	The gorilla’s mouth corners are withdrawn and the lips retracted from teeth and gums. The mouth can be kept closed or widely open. It is a sort of fear grin/grimace
Charging display^O^	The gorilla performs specific postures, movements, facial expressions and vocalizations for threatening the opponent
Chase^O^	The gorilla runs in pursuit the opponent
Crouch/crawl^D^	The gorilla bends all four limbs, presses her/his ventrum to the ground, and tries to travel while in this position, or crouches while sitting by lowering the head, hunching the shoulders, and often covering the head with his/her arm/s
Flee^D^	The gorilla rapidly runs away while she/he is pursued by the opponent
Rigid quadrupedal stance^N^	The gorilla stays in a quadruped position with her/his legs under the trunk. The arms are rigid with elbows turned externally. This posture can represent a threat or a tense situation
Screaming^N^	The gorilla emits an high-pitched, high-volume frightened vocalization
Shelter^D^	The gorilla protects herself/himself from opponent slaps, bites, etc., by putting her/his arms over the head
Staring^N^	The gorilla fixes her/his eyes on a companion with a tense expression for a long period

*N* neutral pattern, *O* offensive pattern, *D* defensive pattern (see the text for definitions of these three categories)

We considered that a play session started when an individual performed any playful pattern (see Table 2) towards a companion and finished when both subjects stopped the interaction (Cordoni et al. 2021). As suggested in the play literature, two consecutive sessions were considered as different sessions if the play interaction stopped for more than 10 seconds (Mancini et al., 2013; Davila-Ross et al., 2015; Cordoni et al. 2016, 2018). In our case, between the first (duration = 35 seconds) and the second (duration = 30 seconds) play session there was a time gap of 35 seconds and between the second and the third session (duration = 270 seconds) the time gap was 20 seconds. Hence, we treated the sequences as three separate playful events. During the play-pause, the silverback and the adult female maintained their spatial proximity and performed self-grooming.

We considered that an aggressive session started when a gorilla directed any aggressive pattern (see Table 2) towards a group mate and usually ended with one of the opponents moving or fleeing.

We classified playful patterns into three categories (see Table 2): (1) offensive (i.e. attack/pursuit playful patterns giving one of the playmates a clear physical advantage over the partner); (2) defensive (i.e. playful patterns by which the player tries to shelter from the playful attack by the partner); (3) neutral (locomotor/acrobatic patterns, possibly involving an object).

To evaluate the asymmetry of each interaction, for each involved party we defined a *play asymmetry index* (PAI) as “the proportion of offensive patterns performed by A towards B *plus* the defensive patterns performed by B towards A” subtracted from “the proportion of offensive patterns performed by B towards A *plus* the defensive patterns performed by A towards B” divided by “the total number of patterns performed by both playmates” (Cordoni et al. 2016, 2018). The PAI ranges from −1 to +1, with main values indicating (i) complete symmetry of the session (zero), (ii) complete asymmetry of the session in favour of A (+1) and (iii) complete asymmetry of the session in favour of B (−1).

$$\mathrm{PAI}=\frac{\text{(}{\mathrm{offensive}}_{\mathrm{A}\to \mathrm{B}}\text{ + }{\mathrm{defensive}}_{\mathrm{B}\to \mathrm{A}}\text{) - (}{\mathrm{offensive}}_{\mathrm{B}\to \mathrm{A}}\text{ + }{\mathrm{defensive}}_{\mathrm{A}\to \mathrm{B}}\text{)}}{\text{(}{\mathrm{offensive}}_{\mathrm{A}\to \mathrm{B}}\text{ + }{\mathrm{defensive}}_{\mathrm{B}\to \mathrm{A}}\text{) + (}{\mathrm{offensive}}_{\mathrm{B}\to \mathrm{A}}\text{ + }{\mathrm{defensive}}_{\mathrm{A}\to \mathrm{B}}\text{) + }{\mathrm{ neutral}}_{\mathrm{A}+\mathrm{B}}}$$

For comparative purposes, outside of the play context we also calculated an aggression asymmetry index (AAI). We considered as (i) “offensive” all patterns performed to threaten/attack/pursue an opponent, (ii) “defensive” all patterns performed to avoid/shelter from an opponent's attack and (iii) “neutral” all patterns not classified as offensive or defensive (see Table 2). AAI was defined as follows: “the proportion of offensive patterns performed by A towards B *plus* the defensive patterns performed by B towards A” subtracted from “the proportion of offensive patterns performed by B towards A *plus* the defensive patterns performed by A towards B” divided by “the total number of patterns performed by both opponents”. Like the PAI, the AAI ranges from −1 to +1, with main values indicating (i) complete symmetry of the session (zero), (ii) complete asymmetry of the session in favour of A (+1) and (iii) complete asymmetry of the session in favour of B (−1).

$$\mathrm{AAI}=\frac{\text{(}{\mathrm{offensive }}_{\mathrm{A}\to \mathrm{B}}\text{ + }{\mathrm{defensive}}_{\mathrm{B}\to \mathrm{A}}\text{) - (}{\mathrm{offensive}}_{\mathrm{B}\to \mathrm{A}}\text{ + }{\mathrm{defensive}}_{\mathrm{A}\to \mathrm{B}}\text{)}}{\text{(}\mathrm{total\, pattern\, performed}\text{)}}$$

We carried out a sequential analysis to evaluate the temporal association between different behavioural patterns. We created a string for each play session by reporting the patterns separated by a break symbol (i.e. |). The resulting string represented the ordered concatenation of patterns as they occurred during each playful interaction. Then, we employed the free open-source software Behatrix 0.9.11 (http://www.boris.unito.it/pages/behatrix; Friard and Gamba, 2020) to analyse the sets of behavioural sequences and organize data into contingency tables. The program generates the code for a flow diagram (Graphviz script) of behaviour-to-behaviour transitions. Via Behatrix, we also calculated the Levenshtein distance, a string metric for measuring the difference between two sequences (Levenshtein 1966; Kruskal, 1999).

## Results

### General and contextual data

In the gorilla colony under study, we collected a total of 590 play sessions, 83 of which involved at least one adult subject. In particular, we recorded three sessions between the silverback (Yaoundé) and the lactating female (Mahmah) and 80 sessions between an adult and an immature subject (number of sessions: 62 Mahmah, 13 Hakuna and 5 Yaoundé). Moreover, we collected 226 aggressive interactions, three of which involved adults. The three aggressions were directed by the silverback towards two adult females (Virunga and Hakuna). No aggression was recorded between the silverback and the lactating female (Mahmah). The spatial proximity between the silverback and adult females accounted for 26% of the proximity bouts (mean dyadic value 1.6 ± 2.1 SD), whereas adult female dyads only accounted for 9.3% of the proximity bouts (mean dyadic value 0.8 ± 3.5 SD).

### Adult playful interactions

On 27 September 2020, we video-recorded three playful sessions between the silverback (Yaoundé) and a lactating female (Mahmah). It was a rainy day and the gorillas spent the whole day indoors, although they had free access to the outdoor enclosure. The group did not receive any energy-rich or special food. No changes were made in the usual routine management. No particular social or environmental stressful event or aggression among group-members occurred during the day. Play sessions occurred during resting time (session #1 occurred at 16:11; session #2 at 16:12; session #3 at 16:15; see video clip in Supporting material), and the two players were in strict proximity or body contact with the immature individuals of the group, including Mahmah’s 3-month old son (Basoko; see Table 1). We did not observe any playful interactions among immature subjects immediately before the Mahmah–Yaoundé play sessions. In all cases, the adult female invited the silverback to play by directing playful contact patterns towards him (i.e. play bite and play pat; see Table 2). It is worth noting that throughout the three adult–adult play sessions, the immature individuals (excluding the newborn, Basoko) tried unsuccessfully to interrupt or join the interaction.

### Play behavioural sequence

We recorded 13 different types of patterns constituting the three play sessions. A total of 37 transitions occurred between behavioural patterns (see Table 3). The Levenshtein distance values ranged from 6 to 20, thus indicating a difference in the composition of the three sessions, particularly between sessions #1 and #2 and #2 and #3 (Table 3).

**Table 3:** Data provided by Behatrix 0.9.11 for behavioural transition and Levenshtein distance in relation to the three play sessions between the silverback (Yaoundé) and adult female (Mahmah)

Number of sequences	3		
Number of different play behaviours	13		
Total number of behaviours	40		
Number of different transitions	26		
Total number of transitions	37		
Levenshtein distance			
	Sequence #1	Sequence #2	Sequence #3
Sequence #1	0	20	6
Sequence #2	20	0	18
Sequence #3	6	18	0

Duration of play sequences in seconds: seq. #1 = 35; seq. #2 = 30; seq. #3 = 270

### Play and symmetry

**Fig. 1:**
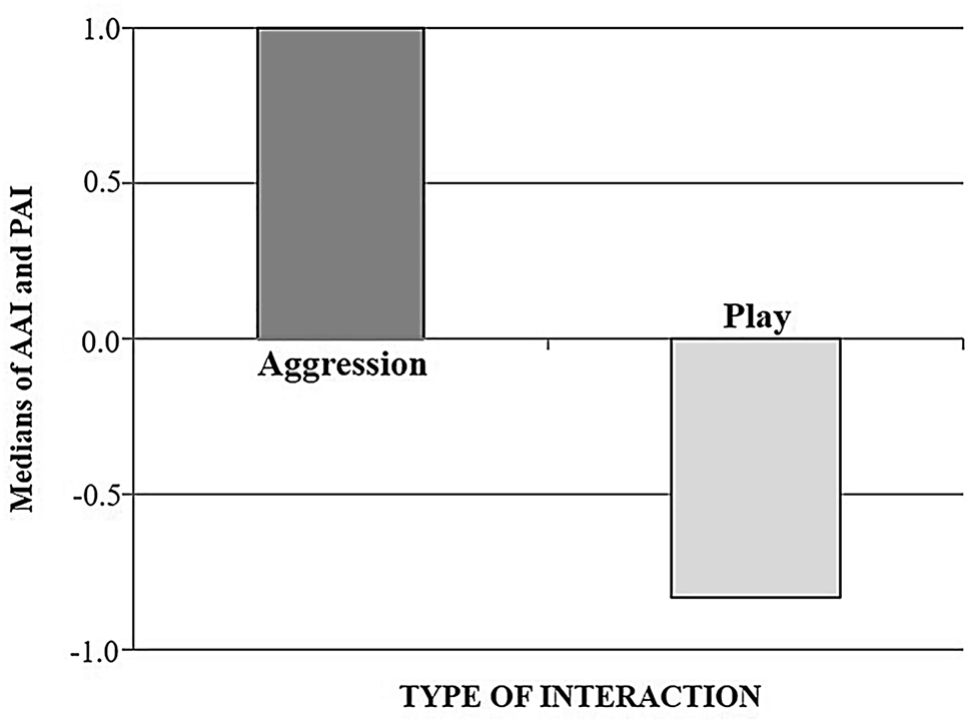
Histogram representing the median values of the AAI and PAI based on aggressive encounters and playful interactions involving the silverback and/or the lactating adult female. The value +1.0 of AAI indicates that during aggression, the silverback performed a higher proportion of offensive and less of defensive patterns compared to the adult female. Conversely, the value around −1.0 of PAI indicates that during play, the adult female performed a higher proportion of offensive and less of defensive patterns compared to the silverback. See the text for definitions of offensive and defensive aggressive/playful patterns

As concerns the symmetry of the session, the medians of PAI (−0.83) and AAI (1.00) indicated two opposite directions (Fig. 1). While in the aggressive context the interaction was completely asymmetric in favour of the silverback, during play the roles of playmates were reversed, with the adult female “dominating” the interaction with the silverback.

### Playful facial expressions

**Fig. 2:**
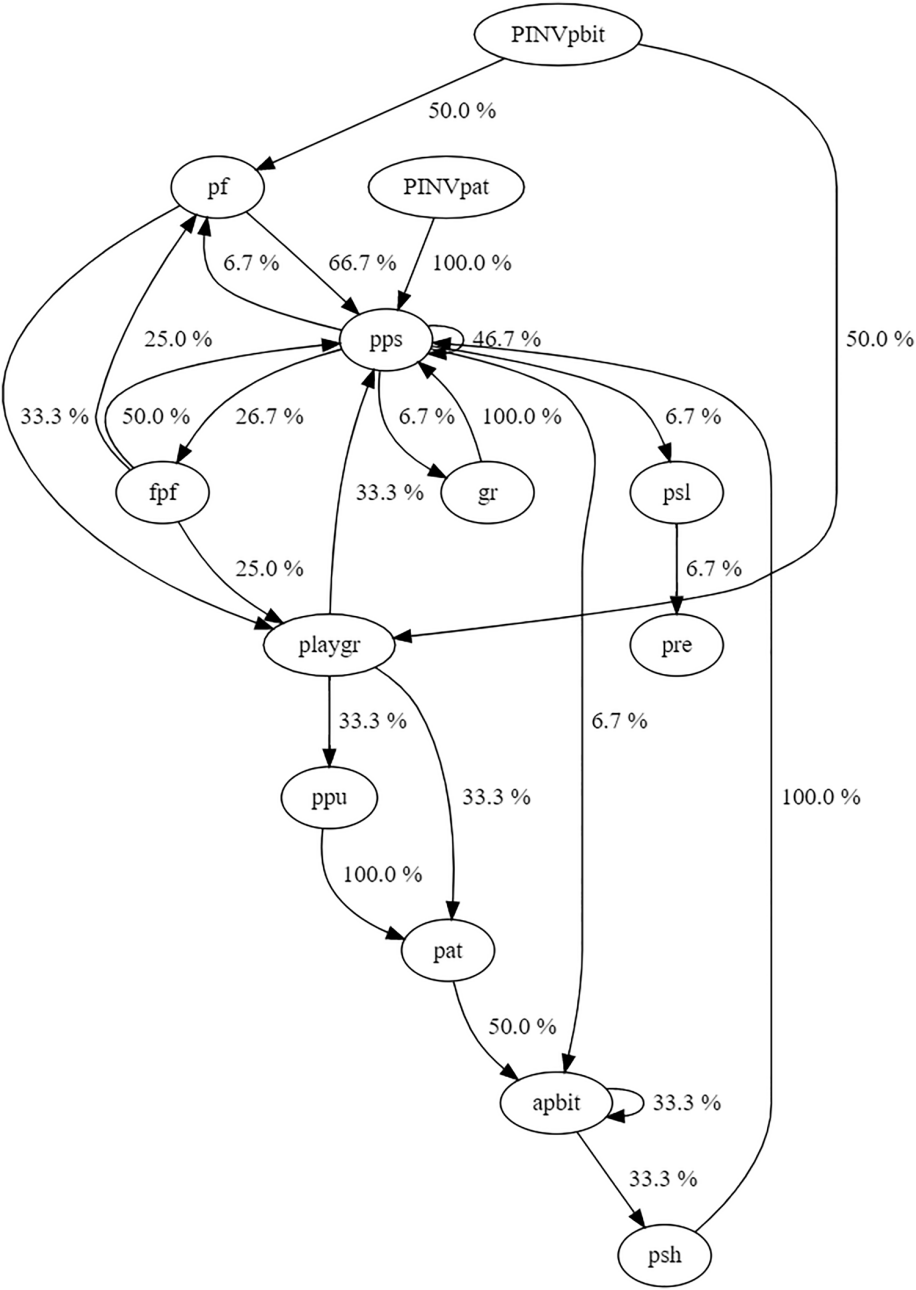
Flow diagram representing behaviour-to-behaviour transitions in the three playful sessions recorded between the silverback and lactating adult female. Legend (see Table 2 for pattern definitions): *abit* attempt play bite, *fpf* full play face, *pat* play pat, *pbit* play bite, *pf* play face, *pinv* play invitation, *plgr* play grab, *pps* play push, *ppu* play pull, *pre* play retrieve, *psh* play push, *psl* play slap

Among the recorded playful patterns, 17.5% were facial expressions mainly performed by the silverback (two play faces, PF, and four full play faces, FPF). The sequential analysis (Fig. 2) showed that PF and FPF performed by both the silverback and the adult female were immediately followed or preceded by a typical playful offensive pattern, that is, play bite (transition occurrence % FPF ↔ play bite = 76.7%; transition occurrence % PF ↔ play bite = 73.4%). In one of the three sessions, namely session #2, the silverback directed a play bite towards the female, and immediately after he performed a FPF while engaging in a face-to-face interaction with Mahmah. Mahmah responded to Yaoundé with a PF and concomitantly directed a play bite towards him (see supporting video material).

### Play and affiliation

During session #2, between two play-biting events the silverback groomed the female for less than 10 seconds (grooming was not part of play; transition occurrence % play bite → grooming 6.67% and grooming → play bite 100%). Generally, during grooming, a gorilla manipulates the fur, extremity or orifice of a companion; grooming may include both manual and oral components (Cordoni & Palagi, 2007). No other grooming bout involving the adults was recorded throughout the duration of our study.

## Discussion

### Presence of adult–adult play

In the current captive study on lowland gorillas, we structurally described three social play interactions between a silverback and an adult female. The novelty of our investigation relies on the report that adult–adult play—although extremely rare—is part of the behaviour of lowland gorillas and can be shown at least under certain circumstances. To our knowledge, no report on play modalities between the silverback and adult females is available in the literature for the study species. Considering that the two subjects involved in the play sessions were born and mother-reared in captivity and thus had no particular history of exploitation in the entertainment industry, we can reasonably exclude that the observed patterns have to be considered as abnormal behaviours. It is possible that the occurrence of such behaviour can be facilitated by the safe social and ecological environment in which the gorillas lived.

In this study, contrary to what is reported in the literature on wild and captive lowland gorillas (Fischer 1983; Stoinski et al. 2003; Stokes 2004; Nakamichi et al. 2014; Klailova and Lee 2014), adult females spent more time in spatial proximity to the silverback than to other females. In this view, the probability of social interaction (including play) between the silverback and adult females was enhanced. However, we observed social play between the silverback and one specific adult female, but not between the silverback and *any* female. Play specifically involved the only lactating female.

Greater concentrations of oxytocin—a neuropeptide hormone—are associated with lactation and milk ejection (Carter et al. 2007; White-Traut et al. 2009). Moreover, even though the relation between oxytocin and social behaviour is highly complex, this hormone could have an effect in creating mother–infant bond, increasing affinitive interactions, forming inter-individual relationships and, in some cases, promoting play (Wallen and Hassett 2009; Insel 2010; Carter 2014; Vanderschuren and Trezza 2014; Numan and Young 2016; Vanderschuren et al. 2016). Hence, in our study the motivation of the female to play might have been increased during lactation. This hypothesis is also supported by the fact that play invitations were always performed by the lactating female towards the silverback. Of course, a much larger sample is necessary to verify this hypothesis. In addition, general levels of play performed by adult females with both other adults and immature subjects should be evaluated before and after the lactation period and compared with levels during lactation. Higher general levels of play during lactation than during non-lactating periods would further support our hypothesis.

Play is considered a by-product of the interaction and rearrangement of different behavioural systems (e.g. aggressive and sexual domains), and its motor patterns resemble those used in these “serious” contexts (Pellis et al. 2019). One of the major risks of play is the misinterpretation of the pattern performed/received by playmates and the consequent escalation of the session into aggression (Pellis and Pellis 1996; Palagi et al. 2016a). In the wild, both mountain and lowland lactating female gorillas receive less aggression by the silverback compared to cycling and pregnant females (Robbins 2009; Breuer et al. 2016). Hence, in our case the risk of the lactating female of receiving an aggressive response by the silverback could be lowered, thus facilitating the start and maintenance of the playful interaction.

In wild mountain gorillas, Grueter and colleagues (2016) recorded two playful interactions between the silverback and adult females in one out of the three groups under observation. The authors did not exclude the possibility that social play could be an effect of food intoxication. In this respect, social play was considered an abnormal, toxin-induced behaviour. Intoxication due to plant consumption was never observed in the study group, and no toxic plants seem to be present in the outdoor enclosure. Therefore, it seems unlikely that social play in our study group was triggered by toxins.

It is also worth noting that during the day in which adult–adult play was observed, gorillas remained mainly inside, and they probably had been expending less energy compared to other ordinary days. The surplus energy theory of play (Barber 1991) proposed that animals can consume the energy in “excess of need” by playing. In this view, adult–adult play could be favoured by an excess of energy subjects have to expend. Nevertheless, in our case the gorilla keepers who have been working with this group for over 20 years told us that they had never observed Yaoundé playing with adult females during “less energy-expending days”.

We can hypothesize that under particular physiological and/or socio-ecological conditions—in our case the possible effects of oxytocin and more frequent spatial proximity—adult–adult play may be present as a rare and “unconventional” part of gorilla social behaviour. Certainly, we cannot exclude the possibility that what we observed is linked to idiosyncratic factors deriving from particular individual life history events that we ignore (e.g. behavioural acquisition caused by prolonged interactions with humans) or to more extroverted personality traits (Racevska and Hill 2017) favouring the play propensity of the lactating female. Moreover, we cannot rule out the possibility that play behaviour in adult gorillas might be an artefact of captivity, although we believe that if this were the case, play behaviour should be much more commonly observed in the captive groups of this species.

### Structure of the play sessions

From a structural point of view, the recorded play sessions showed a degree of variability in the motor patterns performed (13 different types of play patterns; see Table 3) and in the frequency of switching from one pattern to another (37 transitions for a total of 40 patterns performed; see Table 3). In 2011, Burghardt (2011) proposed five criteria for a behaviour to be considered play. In particular, play behaviour (i) does not appear completely functional, (ii) is spontaneous, voluntary and rewarding, (iii) includes patterns exaggerated and modified in their sequences, (iv) comprises repeated but not stereotyped patterns and (iv) is performed in a relatively relaxed context. Our observations are in line with the third and the fourth criteria, which are mainly centred on the behavioural structure of play in terms of pattern repetition and exaggeration.

In our case, we observed that play facial expressions (PF and FPF) mainly occurred immediately before or after an offensive playful pattern. From a classical ethological point of view, PF and FPF are ritualized displays that drive information to the receiver on possible future actions of the play companion. By doing so, such displays reduce the uncertainty concerning the nature of the interaction (Bekoff and Allen 1998; Pellis and Pellis, 1996; Bekoff 2001). Play faces also convey a positive emotional state of playmates (Palagi et al. 2016b, 2019, 2020; but see: Bliss-Moreau and Moadab 2017). It is likely that PF and FPF not only function in communicating the benign intent of the playmate (particularly the silverback), but also allow individuals to potentially share their playful mood.

Finally, the partner roles during play were reversed; indeed, the adult female directed more offensive patterns towards the silverback than vice versa. This strategy is often used by strong, old and dominant individuals for maintaining play with partners having fewer physical abilities and lower hierarchical status (self-handicapping and role reversal; Power 2000; Petru et al. 2009).

In conclusion, this case report confirms that “the absence of evidence does not indicate the evidence of absence”. Although extremely rare, it can be stated that social play between adults is present in lowland gorillas, although its expression may occur in particular individuals living in particular social groups and under particular (if not exceptional) circumstances.

## Supplementary Information

Below is the link to the electronic supplementary material.

Supplementary file1 Video of an indoor playful interaction between the silverback (Yaoundé) and lactating female (Mahmah) of the lowland gorilla colony housed at La Vallée des Singes (France) on 27 September 2020. In the video-clip the salient parts of the playful interaction are described. The two infants (Kouam and Ivindo) and one of the two sub-adult males (Mawete) are in proximity of the players throughout the interaction and unsuccessfully try to join play. Mahmah sits in contact with her newborn (Basoko) during the interaction. Video by Stéphanie Elies. Editing by Giada Cordoni (MP4 125505 KB)

## Declarations

### Research involving human or animal participants

The current research was purely observational, with no manipulation of animals.
